# Seamless Weft Knit Vest with Integrated Needle Sensing Zone for Monitoring Shoulder Movement: A First Methodological Study

**DOI:** 10.3390/ma16165563

**Published:** 2023-08-10

**Authors:** Fei Sun, Zhijia Dong, Yuqin Din, Honglian Cong, Pibo Ma

**Affiliations:** Engineering Research Center for Knitting Technology, Ministry of Education, Jiangnan University, Wuxi 214000, China; feisun0330@163.com (F.S.); dyq0915@126.com (Y.D.); cong-wkrc@163.com (H.C.); mapibo@jiangnan.edu.cn (P.M.)

**Keywords:** wearable electronics, all-textile sensor, one-piece seamless knitting technique, human physiological activity signals

## Abstract

The integration of textile-based flexible sensors and electronic devices has accelerated the development of wearable textiles for posture monitoring. The complexity of the processes required to create a complete monitoring product is currently reflected in three main areas. The first is the sensor production process, which is complex. Second, the integration of the sensor into the garment requires gluing or stitching. Finally, the production of the base garment requires cutting and sewing. These processes deteriorate the user experience and hinder the commercial mass production of wearable textiles. In this paper, we knitted a one-piece seamless knitted vest (OSKV) utilizing the one-piece seamless knitting technique and positioned an embedded needle sensing zone (EHSZ) with good textile properties and electrical performance for monitoring human shoulder activity. The EHSZ was knitted together with the OSKV, eliminating the need for an integration process. The EHSZ exhibited good sensitivity (GF = 2.23), low hysteresis (0.29 s), a large stretch range (200%), and excellent stability (over 300 cycles), satisfying the requirement to capture a wide range of deformation signals caused by human shoulder movements. The OSKV described the common vest process structure without the stitching process. Furthermore, OSKV fulfilled the demand for seamless and trace-free monitoring while effortlessly and aesthetically satisfying the knitting efficiency of commercial garments.

## 1. Introduction

Wearable devices based on textile-based flexible sensors are regarded as promising materials for wearable electronics because they are lightweight [1], have low moduli [2], are comfortable [3], and are durable [4]. As a part of wearable product applications, posture monitoring has attracted sustained interest in the domains of human health, medical aids, posture correction, motion monitoring, and human-computer interaction [5,6,7,8,9,10]. The shoulder joint has a large range of motion, which leads to significant clothing deformation and contributes to a wide range of body movement postures. The study of the monitoring of shoulder joint activity is particularly relevant since the shoulder joint is complex and variable and can be involved in various types of human movement. The pairing of shoulder joint activity with other joints can be employed to identify more human movement postures and thus be utilized in more areas [11,12,13].

There have been several studies on capacitive, resistive, and piezoelectric smart textiles that have demonstrated good performance in monitoring physiological information in the human body [14,15,16]. The principle of these flexible sensors is that the deformation of the fabric caused by stretching or compression leads to a change in capacitance or resistance. Most sensor fabrication processes involve the treatment of the original yarn or fabric through coating, printing, and resin encapsulation. Textile fabrics treated with chemicals typically exhibit better electrical performance, with GF scores reaching 20. However, the use of chemical agents may alter the original comfortability of the textiles [17,18,19,20,21,22]. In terms of practical sensor applications, certain flexible sensors can cause discomfort when they come into direct contact with the skin (e.g., adhesion). Certain flexible sensors, which are integrated into wearable products, can increase the integration process (e.g., through taping, stitching, or embroidery). However, the impact of the integration process on the performance of the sensor is unknown and can be uncomfortable to wear [23,24,25,26,27]. Furthermore, the process and duration of fabrication may increase due to the fabrication of the substrate monitoring garment (e.g., cutting, sewing) [28,29,30,31,32].

In recent years, the one-piece seamless knitting technique has demonstrated significant advantages in wearable textile electronics. The knitted fabric is formed by interlocking coils, which have good tensile recovery and can revert to their initial state after a large strain. The knitted flexible sensing fabric deforms in response to human limb movements, and the deformation variables are transformed into electrical or other readable signals that can be used for human posture monitoring [33,34,35,36]. Furthermore, the technology allows for the positioning of the embedded flexible sensing zone to be knitted while the monitoring product is being knitted in one piece, facilitating a seamless connection between the sensing zone and the monitoring product. This eliminates the need for integration and makes the monitoring product both comfortable and intelligent. Moreover, seam-free and seamless daily wear monitoring is provided with the sensor collected adjacent to the skin and without foreign body sensation [2,37,38,39].

However, there are still certain challenges that prevent the further development of technology. Although the sensor zone can be embedded in commercial garments, it is challenging to achieve unibody formability in commercial garments because of the limited effective strain range of the sensor zone. To prevent the strain range of human activity from exceeding the effective strain range of the sensor zone, it is necessary to avoid pre-stretching of the sensor zone by wearing commercial garments. This further hinders the benefits of universal and seamless manufacturing of commercial garments, which impacts mass production and applications for multiple body types [40,41,42,43,44,45]. Therefore, achieving a balance between good sensor performance and the full formability of commercial garments has become a challenging issue in the development of textile-based sensors.

In this paper, we have employed the process method of floating and loop formation on a weft-knitting circular machine to create a stretchable, comfortable, embedded hanger sensing zone (EHSZ) through yarn addition. This innovative design of EHSZ offers superior sensitivity (GF = 2.23), excellent stability (over 300 cycles), low hysteresis (0.29 s), and good sensitivity to strain measurement at a high stretching capacity of 200%. The combination of the one-piece seamless knitting technology is noteworthy because it allows for the seamless integration of EHSZ in a knitted vest with good aesthetics and comfort, as well as in a one-piece seamless knitted vest (OSKV) with good formability and commodity efficiency. Here, we have built a fully textile wearable health and shoulder movement monitoring system. This system uses the method of establishing a database and then examining the edit distance between two curves to achieve curve pairing. Then, based on the decision tree, the specific posture is identified. The monitor wears the OSKV and detects the limb movements of the human shoulder at different amplitudes by EHSZ, demonstrating good wearable comfort and accuracy.

## 2. Preparation of Experimental Materials

One benefit of the one-piece seamless knitting technique is that the sensor and the monitoring vest can be knitted in one piece, which enables the integration of embedded knitted sensors to be eliminated. This section demonstrates how this technique enables seamless knitting of the monitoring vest, improving comfort while reducing the production process and improving commercial properties.

### 2.1. Knit of One-Piece Seamless Knitted Vest

**Figure 1:**
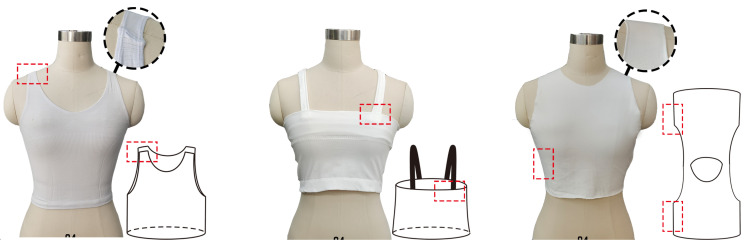
Three types of weft-knitted vest construction (from **left** to **right**, style a, style b, style c).

The one-piece seamless knitting technique was used to knit three of the more common weft knit vest styles from the base garment side. While the vest style “a” eliminates the side seams of a normal vest, the shoulder straps still need to be stitched. Furthermore, the disconnection of the shoulder straps makes integration of the sensor at the shoulder challenging and can impair sensor performance. Despite the elimination of the shoulder seam in vest styles b and c, stitching is still required at the shoulder strap attachment or on the side, which will affect the usability of the sensing area (Figure 1).

**Figure 2:**
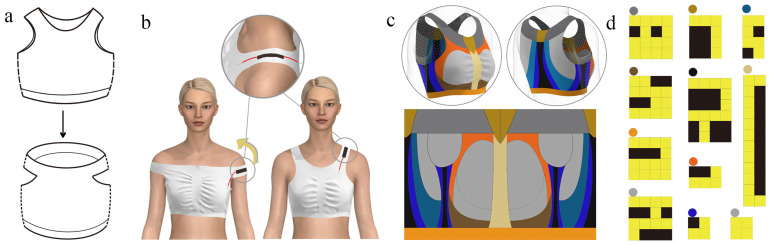
The OSKV straps transformation principle and knitting. (**a**) The direction of the vest straps has been changed from vertical to horizontal. (**b**) Illustration of the horizontal knitted shoulder strap pull up. (**c**) Functional partitioning of 3D vests and 2D templates. (**d**) Fabric structure in different areas.

To address the aforementioned technical problems, this paper presents a weft-knitted base vest for monitoring with a modified shoulder structure. As shown in Figure 2a, the shape of the shoulder straps of a traditional weft-knitted vest longitudinally knitted is transformed into a transversal knit using the one-piece seamless knitting technique to knit a barrel-shaped closed structure, in which the left and right of its transversal shoulder straps are cut, and the transversally knitted straps are stretched to be longitudinally worn (Figure 2b). The resulting transformation of the shoulder strap knitting structure eliminates the shoulder seam commonly found in weft knit vests. This facilitates the integration of the sensing zone.

To achieve the structural transformation of the shoulders, it is necessary to match reasonable functional zoning of the vest, design a suitable tissue structure, and choose the right yarn material. We created a structural, functional partitioning of the weft-knitted vests for monitoring purposes and flattened their 3D modeled partitioning into 2D structural template diagrams (Figure 2c), where different colors represent different partitions. Furthermore, corresponding weft-knitted tissue structures were designed for these partitions (Figure 2d). The yellow in the diagram represents the formation of circles and the black represents the floating line.

**Figure 3:**
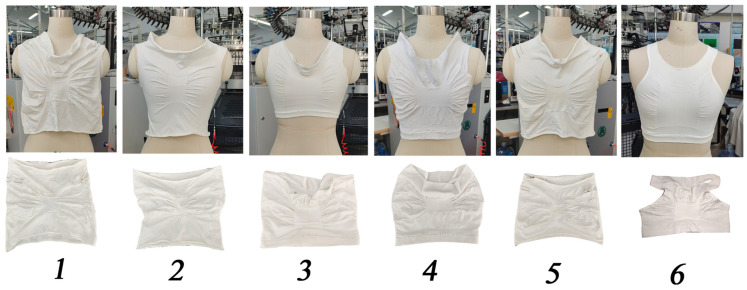
Yarn and tissue construction tests.

We conducted experiments using six different yarn materials and shoulder structures and found that the transformation of the shoulder structure was dependent on the choice of shoulder structure and yarn material (Table 1; yarn parameters for the vest sample). If the shoulder straps are not properly matched, they would not be flexible enough, and there would be a surplus in the front and back centers of the vest after stretching, which reduces comfort (Figure 3). We chose an elastic polyester yarn top yarn and a nylon- and spandex-covered yarn with a three-spaced floating thread shoulder structure.

### 2.2. Knit of Embedded Hanger Sensing Zone

**Figure 4:**
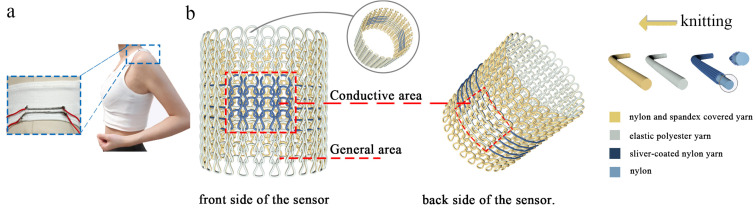
(**a**) Photograph of a one-piece knitted no-integration seamless vest. (**b**) The embedded knitting principle of the ENSZ.

The OSKV can be knitted with a seamlessly embedded knit EHSZ using the inlaid yarn guide technique, which not only eliminates the need for integration but also makes the OSKV comfortable to wear (Figure 4a). The seamless knitting circular knitting machine is a closed cylinder structure knitted along the weft direction. As displayed in Figure 4b, the yarns used are the plain face yarn elastic polyester yarn (shown in white in the picture), the plain bottom yarn nylon- and spandex-covered yarn (shown in yellow in the picture), and the conductive face yarn (shown in blue in the picture). When knitting the non-sensing zone, the plain face yarn is knitted at the same time as the plain base yarn. However, when knitting the sensing zone, the conductive face yarn replaces the plain face yarn in the sensing zone and is knitted at the same time as the plain base yarn, when the plain face yarn is present in the form of a long floating thread on the reverse side of the sensing zone. By replacing the loop with conductive yarn, it is possible to incorporate the integration of an embedded sensing zone (blue area) at any point in the tube.

**Figure 5:**
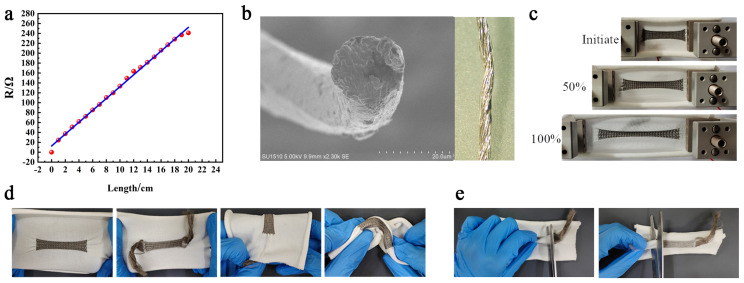
Physical view of the flexible sensor. (**a**) Silver−plated conductive yarn resistance curve with length, (**b**) electron microscope image of a silver−plated nylon conductive yarn. EHSZ clipping processing, (**c**) photographs of EHSZ at increasing starting strain levels of 70% and 133%, respectively, showing its good tensile strain. (**d**) EHSZ’s craftsmanship is front and back, bent and twisted, showcasing its remarkable softness. (**e**) EHSZ clipping processing.

The resistance of the highly elastic silver-plated conductive yarn increases with yarn length (Figure 5a). The optical microscope images of the highly elastic silver-plated conductive yarn are displayed in the inset of Figure 5b, which is made from a 0.2-mm diameter nylon yarn that serves as the foundation for its fibers. The silver layer, which is 99.99% pure, is wrapped around the nylon base material. As indicated in Figure 5c, the strain performance of EHSZ can reach more than 100%. EHSZ offers greater flexibility and a thinner construction than sewing the sensing area onto the base fabric because of its unique embedded process. Figure 5d demonstrates the normal function of the EHSZ despite being stretched, twisted, rolled, and bent. The softness of the EHSZ enables it to assume a variety of shapes and conform to skin deformations during human movement. Figure 5e depicts an image of EHSZ with the white thread on the front side being elastic polyester yarn, the white thread on the reverse side being nylon- and spandex-covered yarn, and the grey thread being high-stretch silver-plated conductive yarn.

### 2.3. The Seamless Knitted of EHSZ and OKSV

**Figure 6:**
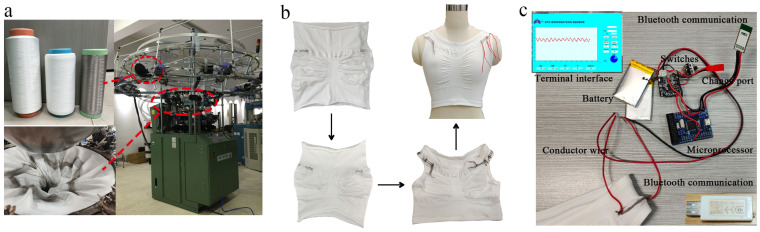
The knitting of OSKV. (**a**) Knitting equipment environment. (**b**) Product Handling Process Flow. (**c**) Wireless transmission of external devices.

The fabric was knitted on a seamless circular knitting machine, an SM8-TOP2 MP2 single cylinder jacquard knitting circular knitting machine (Santoni Knitting Machinery Co., Ltd., Shanghai, China) was used with a cylinder diameter of 15 inches, a needle count of 1344, a gauge of 28 stitches/cm, and 8 tracks (Figure 6a). After dismounting the machine, a one-piece cylinder fabric with EHSZ integrated was obtained. The reserved sleeve arcs were cut to obtain a seamless monitoring vest with integrated EHSZ. A pre-shrinking treatment was performed on knitted samples to make them closer to the sizes of real finished products (Figure 6b). Heat shrinking was conducted at (100 ± 2) °C for 45 min. After that, the samples were dehydrated for 15 min in a dehydrator.

Two sides of the flexible sensing area were left with connectable floating threads. These floating threads were fixed by twisting them together with a 0.08 m^2^ soft silicone conductive wire. The other end of the conductive wire was wrapped around the conductive wire of the data collection system to create a closed series circuit. The dynamic resistance data collection system was equipped with PC desktop application software, which was a testing platform built on the tester’s computer. Compared to a multimeter, this software could display real-time resistance change waveforms. As shown in Figure 6c, the waveform generated was the resistance-time curve. The resistance range could be manually adjusted to achieve optimal observation of the curve. The signal sampling frequency was 10/s, and the resistance collection accuracy was 0.1. The data are finally saved in the form of an electronic spreadsheet.

The OSKV is the pinnacle of excellence for the monitoring product and the sensing area since they are knitted in one piece, free of integration and sewing. The vest can be made without stitching due to the transformation of the shoulder structure. This facilitates the integration of EHSZ in the shoulder knit. The OSKV fully satisfies the need for seamless and seamless monitoring owing to the soft raw material and the knitted structure. EHSZ can be seamlessly integrated into any part of the OSKV to detect information about the body’s shoulder movements. Moreover, it permits a reduction in the production process of the monitored product, resulting in efficient production with lower costs.

## 3. Test Results and Analysis of EHSZ

### 3.1. Sensing Performance of the EHSZ

**Figure 7:**
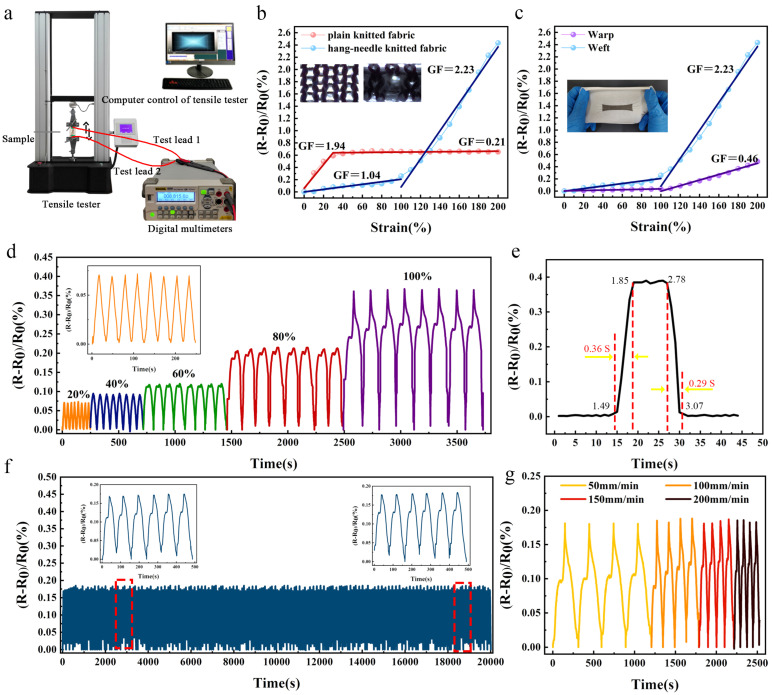
The strain sensing performance of the fabric sensor. The Δ*R*/*R*_0_ of the sensor under cyclic stretching. (**a**) Photograph of the testing instrument system. (**b**) Resistance change as a function of strain at a stretching rate of 100 mm/min. (**c**) Relative resistance changes of the EHSZ strain sensor as a function of the applied strain in the longitudinal and transverse directions, respectively. (**d**) Dynamic responses under a repeated strain of 20%, 40%, 60%, 80%, and 100% (100 mm/min) for 8 cycles. (**e**) The real-time Δ*R*/*R*_0_ of the sensor subjected to a fast-speed (500 mm/min) stretching and releasing at 5% strain. (**f**) Stability testing under 80% strain at 200 mm/min for 300 cycles. (**g**) Cyclic resistance changes under 80% strain at a speed of 50, 100, 150, and 200 mm/min.

Figure 7a displays the sensor test platform. Six tests were performed on the sample and the average was calculated. A RIGOL DM3068 digital multimeter (Beijing Puyuan Precision Technology Co., Ltd., Beijing, China) was used to measure the resistance value of the sample at all stages, the maximum accuracy of the digital multimeter was 6.5 digits, the test rate was 10 k readings per second, and the accuracy was 0.0035% of the open circuit voltage (OCV). A YG028 fabric strength machine (Ningbo Textile Instrument Factory, Ningbo, China) was used to simulate stretching.

As shown in Figure 7b, the strain resistance of the sensor with weft stretching is divided into two stages for both the flat needle and the hanging needle structures. The first phase for the flat needle structure linearly increases (GF = 1.94). However, when stretched further beyond the effective strain range, the second phase no longer exhibits a significant change in resistance (GF = 0.21). The hanging pin structure linearly increases in the first phase (GF = 1.04) and sharply rises in the second phase (GF = 2.23), which reflects the larger effective strain range of EHSZ (200%). The strain sensitivity GF of the sensor is an important parameter that characterizes the sensing performance of the sensor, which refers to the strain-resistance effect of the conductor. It is defined as follows: the higher the sensitivity of the sensor, the better the sensing performance (in the equation: Δ*R* is the corresponding resistance change of the strain sensor, *R*_0_ is the initial resistance in the unstretched state, *ε* is the strain in the stretching direction of the sensor.)

$$GF=\frac{\Delta R/R_{0}}{\varepsilon }$$

As shown in Figure 7c, EHSZ was stretched in the latitudinal and radial directions to examine its strain resistance variation characteristics. It was observed that the sensing zone was more sensitive when stretched along the latitudinal direction and had a GF of 0.46 when radially stretched. This characteristic must be considered in subsequent integration, and the latitudinal stretching of the sensor must follow the direction of the large body strain.

Figure 7d shows the stable sensing responses under various cyclic stretching strains. Furthermore, the linear relationship between Δ*R*/*R*_0_ and the applied strain is evident. The response time to stimuli is crucial for the strain sensors to detect strains in real time. On the application of a small loading strain to the sensor and subsequently unloading at a fast rate, the loading and unloading durations of the sensor are observed to be 0.36 and 0.29 s, respectively (Figure 7e), demonstrating a low latency.

The material selection and structural design of the fabric sensor must meet the requirements of wearable applications. Hence, the stability of Δ*R*/*R*_0_ during cyclic stretching and the sensing performance of the sensor after different treatments to simulate real-world uses were investigated to confirm its reliability and durability.

The soft, nylon- and spandex-covered yarn ground fabric positively contributes to the steady sensing response of the sensor. The fact that Δ*R*/*R*_0_ is constant for 10,000 stretching cycles (Figure 7f) demonstrates the high durability and stability of the strain sensor. As shown in Figure 7g, the sensor maintains a constant Δ*R*/*R*_0_ during cyclic stretching at different rates.

### 3.2. Structure and Working Mechanism of EHSZ

**Figure 8:**
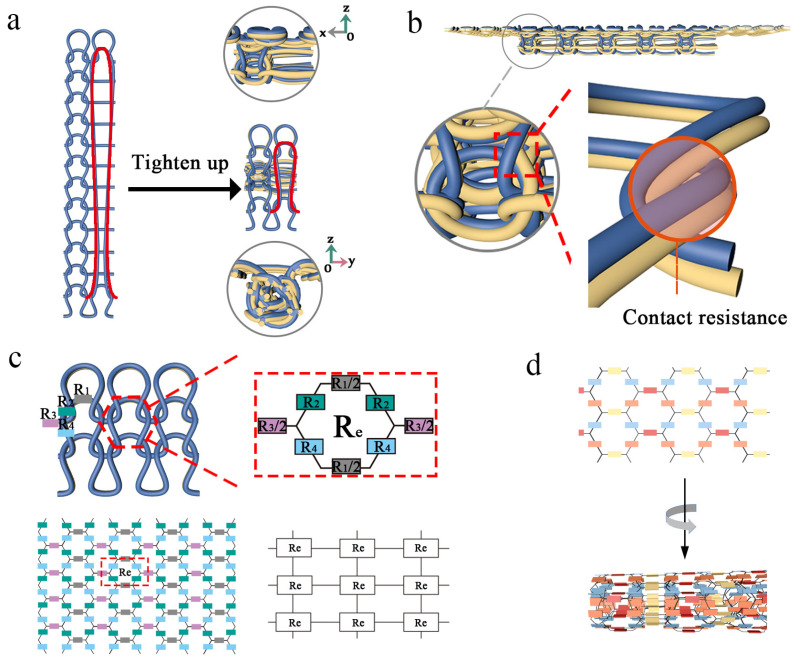
Demonstration of the working principle of the EHSZ. (**a**) The structural process of the EHSZ. (**b**) Contact resistance. (**c**) Equivalent resistance model for a flat pin structure. The blue color in the image represents the conductive top yarn, the yellow color represents the normal bottom yarn, and the red color represents long loops. (**d**) Equivalent resistance model of the hanger pin structure.

The principle for forming the structure of EHSZ is depicted in Figure 8a. The conductive top yarn and the normal bottom yarn are knitted together as an additional yarn in the sensing area, with multiple rows of floating threads in the longitudinal direction. This special shrinking process makes the EHSZ stronger than the normal plain needle structure. The deformation of the coil structure due to the force-induced deformation of the knitted fabric, which alters the total fabric resistance, serves as the sensing mechanism of a knitted strain sensor. This change in strain resistance is directly related to the sensitivity of the sensor. The change in strain and sensor resistance is influenced by length resistance, coil transfer, contact resistance, and superimposed resistance.

Figure 8c, which presents a two-dimensional planar resistance model, is the resistance hexagonal model of the most commonly used sensor with a flat needle structure. The change in transfer of the coil structure in the two-dimensional direction during stretching is a significant factor contributing to the change in resistance. The Figure 8b shows the contact resistance of the EHSZ sensor, Figure 8d shows that the resistive hexagonal model of the EHSZ sensor prepared in this paper produces a three-dimensional curl on top of the two-dimensional plane, which significantly increases the number of superimposed and contact resistances inside the sensing zone. This is due to the tightly curled structure obtained, with coils in close contact with each other and coils squeezed against each other. During stretching, EHSZ exhibits a longer strain range and better sensitivity than the flat-pin structure.

## 4. Real-time Monitoring of Human Respiration

**Figure 9:**
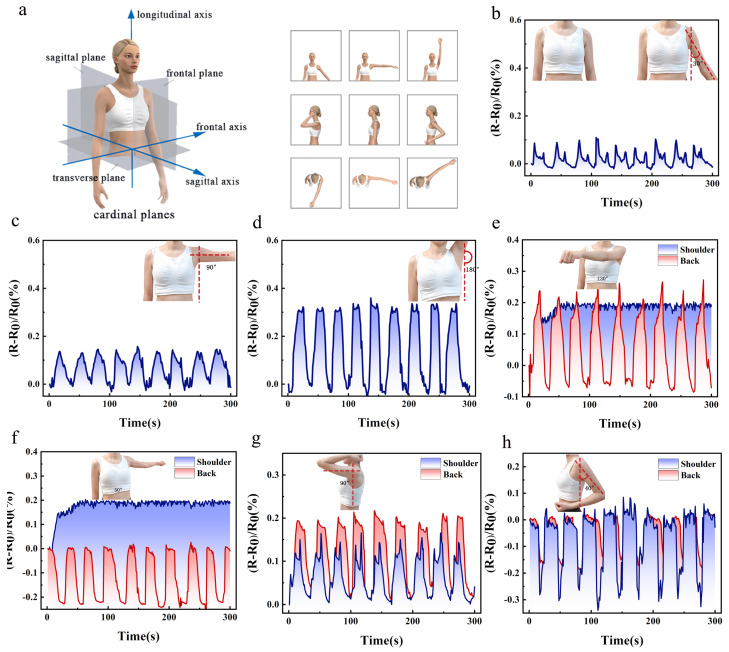
Real−time monitoring of human physiological activities using OSKV. (**a**) The basic movements of the human shoulder in three anatomical planes. Electrical signal output from the same joint at different swing angles: (**b**) 30° in the coronal plane, (**c**) 90° in the coronal plane, (**d**) 180° in the coronal plane, wave patterns of signals picked up by sensors during. (**e**) Inward to 130° in the horizontal plane, (**f**) outward to 50° in the horizontal plane, (**g**) 90° in the sagittal plane, (**h**) 40° in the sagittal plane.

As illustrated in Figure 9a, the human body can move along or parallel to the fundamental plane, and the movement of the shoulder can be defined as sagittal, coronal, and horizontal. The three fundamental planes allow for the identification of the virtual model wearing OSKV. The frontal plane vertically divides the body into two parts, the front and the back. The shoulder laterally moves in this plane, choosing a 30°, 90°, or 180° stance in the coronal plane. The sagittal plane vertically divides the body into two parts. In this plane, the shoulders move forward and backward, in a 90°, initial, 40° sagittal forward flexion, and posterior extension position. The transverse plane horizontally divides the body into upper and lower parts, and the horizontal plane is chosen for 130° of inversion, initial, and 50° of abduction.

The initial investigation involved the analysis of the stance and strain resistance curves of the human shoulder during coronal plane motion. As shown in Figure 9b–d, when the human shoulder moves in the coronal plane, there is hardly any discernible pattern of resistance change in the back sensing zone. The resistance change values increase with increased movement in the shoulder sensing zone, which significantly fluctuates. When the shoulder joint was lifted to 30° from 0° in the coronal plane, and this movement was repeated, the wave of resistance change in the shoulder sensing zone approximated 0.08. The double wave of the resistance curve was due to a delay in the response when the sensing zone was retracted. The shoulder sensing zone resistance wave crest approached 0.13 when the shoulder joint was raised from 0° to 90° in the coronal plane, and this maneuver was repeated. However, the trough was insignificant. When the shoulder joint was raised from 0° to 180° in the coronal plane, and this maneuver was repeated, the wave crest in the shoulder sensing zone reached 0.32, which was the largest fluctuation range of resistance values of the three maneuvers. Based on the peak values of resistance that could be reached, a preliminary identification of the three movements of the shoulder joint in the coronal plane was made.

The stance and strain resistance curves of the human shoulder during movement in the horizontal plane were analyzed. As shown in Figure 9e,f, the initial stance in the horizontal plane is with the arm horizontally extended, which is a 90° posture of lifting on the coronal plane. Meanwhile, during the lifting process, the rate of change of resistance in the sensing area of the shoulder reaches 0.2. The fluctuation value of the sensing area of the shoulder is extremely small and stable when the human shoulder moves 130° inward and 50° outward. At this point, further discrimination of posture is required based on the value of the change in resistance in the sensing zone of the human back. When the shoulder joint is at 0° in the horizontal plane, the sensing zone of the back is driven by the scapula with a smooth value of 0. When the shoulder joint is at 0° inward to 130° in the horizontal plane, the sensing zone of the back is driven by the scapula with a rate of change in resistance of 0.2. When the shoulder joint is at 0° outward to 50° in the horizontal plane, the sensing zone of the back is vacant in the middle of the spine with a certain amount of space, and the rate of change in resistance of the sensing zone of the back is −0.2.

The analysis of the stance and strain resistance curve of the human shoulder during sagittal plane movement was conducted. As indicated in Figure 9g,h, the initial posture in the sagittal plane is the 0° posture in the coronal plane, which is the normal upright posture of the human body. The movement of the shoulder in the sagittal plane causes fluctuations in the shoulder and back sensing zones, which must be combined with the fluctuation curves of the two sensing zones for comprehensive identification. As the change in resistance values of the back sensing zone in the horizontal and sagittal planes are not very different, identification errors will be generated, which must be combined with the shoulder sensing zone for further examination. When the shoulder joint is flexed forward from 0° to 90° in the sagittal plane, the shoulder sensing zone fluctuates to 0.1. Due to the upward movement of the shoulder joint, the binding force of the shoulder belt increases, the stretching of the shoulder belt becomes larger, and the shoulder sensing zone fluctuates to 0.1. Meanwhile, the back sensing zone fluctuates to 0.2 due to the movement of the scapula. When the shoulder joint is extended backward from 0° to 40° in the sagittal plane, the shoulder sensing zone decreases due to the downward movement of the shoulder joint. Due to the inward retraction of the scapula, the binding force of the shoulder belt during recovery causes the shoulder sensing area to fluctuate to −0.3 and the back sensing area to fluctuate to −0.2.

**Figure 10:**
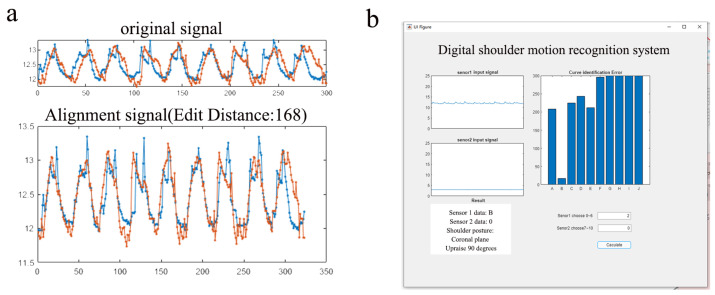
(**a**) EDR algorithm for edit distance fitting, Blue: Dataset H, Red: Dataset K. (**b**) GUI interface for the digital shoulder movement recognition system.

The study of an algorithm to determine the similarity of two curves belongs to the field of curve similarity or curve matching. This section employs an algorithm—the edit distance on real sequence—to perform trend fitting of data curves, discerning the shoulder movements corresponding to different curves based on the edit distance values of the two sets of motion curves. Figure 10a shows the results of the aligned signals for the two datasets. The smaller the edit distance, the more similar the curves are, and the more accurate the identification is, even with a small difference in the rate of change but different curve fluctuations.

**Figure 11:**
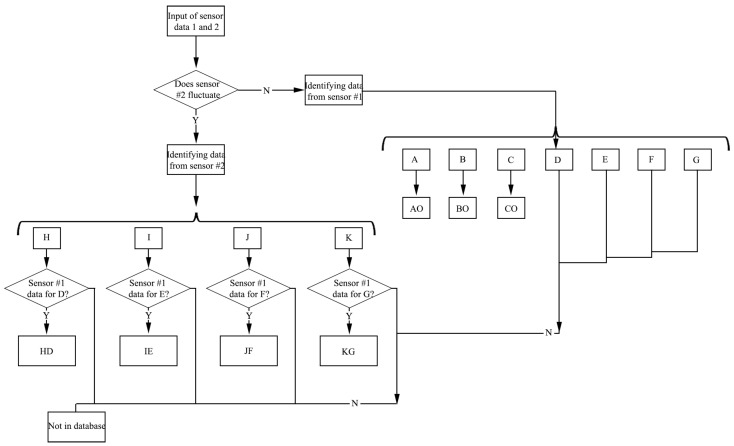
Decision trees for shoulder movements.

Seven datasets of A, B, C, D, E, F, and G about sensing zone 1, and four datasets of H, I, J, and K about sensing zone 2 are created in the system (Table 2). A set of acquisition curve signals identified by a single sensing area is put into the dataset and identified with it in turn. A fit of the two sets of curves is performed to compare the relative distance between the two sets of curves and to find the two curves with the smallest relative distance in the dataset. This can be regarded as having the smallest identification error or even a complete overlap to build a decision tree (Figure 11).

To perform the neural network modeling and data analysis, MATLAB version 2021a was used as the working environment. Figure 10b shows the final built digital shoulder action recognition system with a GUI interface.

## 5. Conclusions

In this paper, we knitted a one-piece seamless sports vest (OSKV) using the one-piece seamless knitting technique and positioned an embedded knitted hanger sensing zone (EHSZ) with good textile and electrical properties for the monitoring of human shoulder movement. The following conclusions were drawn: converting the shoulder straps of weft-knitted shaped vests from longitudinal to transverse knitting eliminates the shoulder seams commonly found in weft-knitted shaped vests and makes it easier to integrate sensors. The sensor in the hanging needle construction matches the performance of the sensor in use and enables effective monitoring compared to plain knit fabrics. Analyzing the Edit Distance on Real sequence of the resistance curve ensures precise posture recognition. It is anticipated that in the future, smart products such as EHSZ will be perfectly incorporated into our technological lives in a more comfortable and sensory-free way.

## 6. Limitations and Future Direction

We will conduct research from the following three perspectives. First, we will try to place the monitoring vest on individuals with different body types to observe the changes in strain resistance and examine the impact of body type on the data. Second, we will wash and dry the monitoring vest and immerse it in sweat to simulate wearing conditions on the monitoring data. Third, we will add additional sensing areas to other parts of the body and collect data on the shoulder movement posture in reference to specific shoulder rehabilitation or treatment actions. We will also conduct multiple measurements and establish a confusion matrix to examine the accuracy of the recognition system.

## Figures and Tables

**Table 1:** Yarn parameters for the vest sample.

Sample	Base Yarn1	Base Yarn2	Top Yarn	Fabric Construction at the Shoulders
1	Nylons 77 dtex	Nylons 77 dtex	Polyester 77 dtex	1 needle floating thread
2	Nylon and spandex covered yarn 56 dtex	Nylons 77 dtex	Polyester 77 dtex	1 needle floating thread
3	Nylon and spandex covered yarn 56 dtex	Nylon and spandex covered yarn 56 dtex	Elastic polyester yarn 56 dtex	2 needle floating thread
4	Nylon and spandex covered yarn 56 dtex	Nylons 77 dtex	Polyester 77 dtex	3 needle floating thread
5	Nylon and spandex covered yarn 56 dtex	Nylon and spandex covered yarn 56 dtex	Polyester bi-component fibres 56 dtex	2 needle floating thread
6	Nylon and spandex covered yarn 56 dtex	Nylon and spandex covered yarn 56 dtex	Elastic polyester yarn 56 dtex	3 needle floating thread

**Table 2:** The shoulder movements correspond to the sensor number.

Anatomical Plane	Movement	Angle	Sensing Zone 1 on the Shoulder	Sensing Zone 2 on the Back
Coronal plane	Upraise	30°	A	O
	Upraise	90°	B	O
	Upraise	180°	C	O
Horizontal plane	Inwards	130°	D	H
	Outwards	50°	E	I
Sagittal plane	Back extension	40°	F	J
	Forward flexion	90°	G	K

## Data Availability

Not applicable.
